# Seeing sadness: Comorbid effects of loneliness and depression on emotional face processing

**DOI:** 10.1002/brb3.2189

**Published:** 2021-05-30

**Authors:** Survjit Cheeta, Joseph Beevers, Sophie Chambers, Andre Szameitat, Chris Chandler

**Affiliations:** ^1^ Department of Life Sciences Centre for Cognitive Neuroscience College of Health Medicine and Life Sciences Brunel University London Uxbridge UK; ^2^ Oxford Health NHS Foundation Trust Warneford Hospital Oxford UK; ^3^ School of Social Sciences London Metropolitan University London UK

**Keywords:** comorbidity, depression, emotional face processing, loneliness, young adults

## Abstract

**Background/Objective:**

Loneliness and depression are highly comorbid, and both are associated with social processing deficits. However, there is a paucity of research aimed at differentiating emotional face‐processing deficits that are comorbid to loneliness and depression versus those attributable to loneliness or depression only.

**Methods:**

502 participants were recruited and screened for loneliness (UCLA Loneliness Scale) and depression (Beck Depression Inventory). Of those, seventy‐seven took part in a fully crossed 2 (loneliness; low/high) * 2 (depression; low/high) factorial between‐subjects design study to assess individual and comorbid effects of loneliness and depression on a computerized morphed facial emotion processing task.

**Results:**

Comorbidity was confirmed by a significant positive correlation between loneliness and depression. On the emotion processing task, loneliness was associated with an increased accuracy for sad faces and decreased accuracy for fearful faces and depression with decreased accuracy in identifying happy faces. Comorbid loneliness and depression resulted in an increased misattribution of neutral faces as sad, an effect that was also seen in those who were either only lonely or only depressed.

**Conclusion:**

This if the first study to tease out comorbid versus independent effects of loneliness and depression on social information processing. To the extent that emotional biases may act as risk factors for detrimental outcomes, our findings highlight the importance of treating both loneliness and depression.

## Graphical abstract

Loneliness and depression are highly co‐morbid and both are associated with basic social processing deficits. However, there is a paucity of research aimed at differentiating emotional face processing deficits that are attributable to loneliness versus depression only. Using a novel fully crossed experimental design, the present study aims to tease out individual effects that are due to loneliness versus depression. This will enhance our understanding of loneliness and depression.

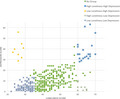

## 1 INTRODUCTION

Loneliness, defined as a discrepancy between the desired versus the perceived quality of social relationships (Peplau & Perlman, 1982), is highly comorbid with depression (Cacioppo et al., 2006; Liu et al., 2016; Mahon et al., 2006). As well as being an independent risk factor for depression (Jaremka et al., 2013), longitudinal studies have reported that loneliness also increases existing depressive symptomatology (Cacioppo, Hawkley, et al., 2006; Cacioppo, Hughes, et al., 2006). Furthermore, a meta‐analysis of 100 published studies found social support acted as a protective risk factor against depression (Gariepy et al., 2016). Both loneliness and depression have independently been associated with impairments in the processing of social information, a skill that is critical for communication success, social functioning, and maintaining interpersonal relationships (Adolphs, 2003).

Lonely individuals are more responsive to images of unpleasant social threats, that is, instances of social rejection by others (Cacioppo et al., 2016). In real‐life settings, loneliness was associated with increased eye gazing toward unfamiliar partners, with findings also revealing that lonely individuals have difficulties in interpreting eye gaze cues, a finding which correlated with reduced gray matter in the left posterior temporal sulcus (Kanai et al., 2012). Using dynamic stimuli, no relationship between loneliness and emotional recognition or the recognition of micro‐expressions was found (Lodder et al., 2016). However, individuals with fewer friends were more accurate in identifying negative and positive facial emotional cues (Gardner et al., 2005), lending support to the theory that belonging deficits lead to higher monitoring in social situations. In a study with adolescents (mean age 13), when depression and social anxiety were controlled for, lonely individuals were more accurate at identifying the negative emotions of sadness and fear (Vanhalst et al., 2017).

Mood‐congruent biases in facial emotional processing have consistently been reported in depression (Elliott et al., 2011). A general decrease in sensitivity to emotional faces has been found (Csukly et al., 2009; Leppänen, 2006) with depression promoting negative biases in identifying facial emotions. Depression has also been associated with greater sensitivity to sad faces compared with other emotions and greater response bias toward misattributing neutral faces as sad (Gilboa‐Schechtman et al., 2002). Depressed patients also show reduced sensitivity toward happy faces, needing more intense expressions to correctly identify this emotion (Chan et al., 2009; Joormann & Gotlib, 2006).

The magnitude of the correlation between loneliness and depression has raised questions about their conceptual and functional separation (Cacioppo, Hawkley, et al., 2006; Cacioppo, Hughes, et al., 2006; Wilson et al., 2007). However, questionnaire items measuring loneliness and depression load on different factors highlighting separation (Cacioppo et al., 2006a; Cacioppo, Hughes, et al., 2006). Longitudinal studies also suggest that loneliness predicts increased depressive symptomatology over a one‐year period but depression does not reciprocally predict increased loneliness (Hawkley et al., 2006). Further, experimentally induced loneliness through hypnosis was associated with increases in depression and perceived stress (Cacioppo et al., 2006a, 2006b). However, as the effects of loneliness or depression on emotional processing have only been investigated in separate studies, it has been difficult to tease out any differences in emotional processing that are attributable to loneliness versus depression in a comorbid group. To address this specific question, a fully crossed 2 (loneliness; low/high) * 2 (depression; low/high) factorial between‐subjects design involving healthy and lonely participants with and without depression was conducted to allow for the assessment of individual effects of loneliness and depression as well as their combined impact on a forced‐choice facial emotion recognition task. As neurobiological theories of depression suggest mood‐congruent information processing (Willner et al., 2013), it was hypothesized that depressed participants would show biases toward negative emotions and away from positive emotions. Theoretical accounts of loneliness offer different perspectives on the effects of loneliness on social perception. Some suggest that loneliness is associated with increased perception of threat, which would lead to heightened perception of negative/threatening stimuli, whereas belonging deficit theories of loneliness suggest a general increase in levels of social monitoring. Based on the relationship between loneliness and depression, we predicted loneliness to be associated with a negative processing bias which would be heightened in those comorbid for depression and loneliness.

## 2 METHODS

### 2.1 Participants and design

Brunel University London College of Health Medicine and Life Sciences Research Ethics Committee approved this research. Participants were recruited through social media, the Division of Psychology participation pool, and word of mouth. First‐year psychology undergraduates were offered one research participation credit for completing the first part of the study (questionnaire measures of loneliness and depression) and if they were eligible, two further credits to take part in the second part of the research (emotional face‐processing task); no other incentives were offered. All participants were required to be aged 18 or over. To investigate the relationship between loneliness and depression in the first part of the study, a correlational study design was used. For the second part of the study, a fully crossed 2 (loneliness; low/high) * 2 (depression; low/high) factorial between‐subjects design was used. The Beck Depression Inventory‐II scores (Beck et al., 1996) were used as the measure of mood and low depression defined as a score in the range of 0–13, and high depression as a score between 25 and 63 (Beck et al., 1996; Roelofs et al., 2013). Loneliness was measured using the UCLA loneliness scale version 3 (Russell, 1996), with low loneliness being assigned to those scoring between 20 and 35 on the UCLA, and high loneliness for those who scored 60 and 80 (Russell, 1996). A total of 502 participants (182 male mean = 27.81, *SD* = 9.51 and 320 females, mean = 29.64, *SD* = 13.23, range 18–70 years) were recruited to the study who all completed the BDI and the UCLA loneliness scale. From this initial screening, participants were eligible to take part in the second part of the study (emotional recognition task) if their scores were in one of the four experimental groups, that is, low loneliness/low depression, low loneliness/high depression, high loneliness/low depression, and high loneliness/high depression. One hundred and twenty‐six participants screened for low loneliness/low depression (controls), and of these, 35 were randomly selected for completion of the emotional processing task (15 Male, 20 Female). A further twenty‐three participants screened for high loneliness/high depression of which twenty‐one (11 Male, 10 Female) completed the emotional processing task. The most difficult experimental groups to recruit were high loneliness/low depression or low loneliness/high depression. All 10 participants with low loneliness/high depression, that is, depression only (4 Male, 6 Female) and all 11 participants with high loneliness/low depression, that is, loneliness only (4 Male, 7 Female) completed the emotional processing task. 2 * 2 factorial ANOVA's were conducted on age, gender, education level, and NART scores and no significant differences were found (Table 1). The remaining 332 individuals were not eligible for participation as they fell outside of one or more of the scores necessary for grouping participants.

**TABLE 1:** Participant characteristics

	HL/HD (*n* = 21) Mean (*SD*) Male: 11, Female: 10	LL/HD (*n* = 10) Mean (*SD*) Male: 4, Female: 6	HL/LD (*n* = 11) Mean (*SD*) Male: 4, Female: 7	LL/LD (*n* = 35) Mean (*SD*) Male: 15, Female: 20	Statistics
Age	28.24 (12.52)	29.30 (13.12)	30.64 (11.96)	28.63 (12.79)	*F* (3,76) = 0.09, *ns*
NART	101.38 (6.16)	98.20 (5.05)	103.27 (4.19)	99.91 (6.12)	*F* (3,76) = 1.65, *ns*
BDI‐II	38.10 (9.94)	38.20 (7.87)	4.00 (3.03)	3.94 (3.27)	*F* (3,76) = 175.92, *p* < .001
UCLA	64.10 (3.61)	26.40 (1.89)	61.91 (2.07)	28.60 (2.83)	*F* (3,76) = 1,034.93, *p* < .001

Note

Statistics are one‐way analysis of variance with a between –subject group factor (Hl/HD, LL/HD, HL/LD, LL/LD).

Abbreviations: BDI‐11, Beck depression inventory‐11; HL/HD, high loneliness and high depression; HL/LD, high loneliness and low depression; LL/HD, low loneliness and high depression; LL/LD, low loneliness and low depression; NART, National Adult Reading Test; UCLA, UCLA loneliness scale version 3.

### 2.2 Procedure

The present study was conducted in two stages. Initial screening of participants for loneliness and depression was conducted online via Qualtrics® Survey Software (Qualtrics®). Following online reading of the participant information sheet, participants were required to give their informed consent using a tick box approach, which confirmed they were aged 18 or over. All participants then completed the Beck Depression Inventory (BDI‐II: Beck et al., 1996) and the UCLA loneliness scale (Russell, 1996) Participants were informed that dependent on their scores in the first stage of the study, they may be invited to take part in the second stage of the study. If participants were eligible, they were invited by email to take part in the second stage, which was scheduled within 2 weeks of the online screening. The second stage was conducted face‐to‐face in the laboratory, and participants were required to complete the National Adult Reading Test (NART; Nelson & Willison, 1991) and the emotional face‐processing task.

### 2.3 Materials

#### 2.3.1 Subjective measures

##### Beck Depression Inventory‐II

The BDI‐II (Beck et al., 1996) consists of 21 sets of four statements where participants must choose which statement in each set best describes how they have been feeling over the past 2 weeks. The statements establish current mood, personal beliefs in the present and about the future, general functionality, and self‐perception in relation to others. For each statement, there are four response options (0–3) with increasing scores suggesting greater depression. The sum of scores from all twenty‐one statements (ranging from 0 = 63) determines the depression score, with scores of 0–13 indicating minimal depression, 14–19 mild depression, 20–28 moderate depression and 29–63 severe depression (Roelofs et al., 2013). 118 studies, which measured the applicability of the BDI‐II, showed good structural validity (Wang & Gorenstein, 2013) and high test–retest reliability (α = 0.83–0.96).

##### UCLA loneliness scale version 3

The UCLA loneliness scale (Russell, 1996) comprises 20 questions, which participants must respond to with either “Never,” “Rarely,” “Sometimes,” or “Always.” There are both positive and negative questions relating to personal feelings about isolation, relationships, social situations, support networks, and specific personal traits. Total score can range from 20 to 80, with a higher score indicating a greater perception of loneliness. The test–retest reliability of this measure is high (*α* = 0.89–0.94), and strong internal consistency and construct validity have been found (Russell, 1996).

##### National adult reading test

The NART (Nelson & Willison, 1991) is a list of 50 words used in the English language, which all have irregular spellings, and are, therefore, difficult to pronounce. This tests adult vocabulary, thus providing an indication of premorbid intelligence. Participants are required to read the words aloud, one by one, and their score equates to the number of correct pronunciations. This test is widely used and is considered an adequate measure of verbal intelligence (Bright et al., 2002; Crawford et al., 2001). This test was used to ensure participants were able to read.

### 2.4 Objective measures

#### 2.4.1 Facial emotion recognition task

This computer task asked participants to identify which emotion, either happy, sad, fear, anger, disgust or surprise, was represented by the face presented on the computer screen. The faces were acquired from (Ekman & Friesen, 1971) pictures of facial affect series. These varied by actor, emotion presented, and the intensity of the emotion shown on the face, either 0% (neutral), 25%, 50%, 75%, or 100%. The task was created and presented on a PC via E‐Prime® by Psychology Software Tools (2.0, Psychology Software Tools).

When completing the task, participants were first provided with a trial set of faces to gain familiarity with the process before taking part in the experimental set. For each individual stimulus, the participant was first shown a fixation cross in the center of the screen, followed by the stimulus image for 500 ms, and then, a visual mask which also lasted 500 ms. The six emotions were subsequently displayed on the screen in six boxes to form a circle of options, and participants were instructed to use the computer mouse to select the emotion, which corresponds to the face they had just seen. In total, 144 individual faces were randomly presented, with the entire task taking 15 min. Accuracy of emotional identification and reaction times, plus nominated misattributed emotional category when perceiving neutral faces, were recorded.

### 2.5 Statistics

All data were analyzed using IBM SPSS Statistics version 23 for Windows. The relationship between loneliness and depression was analyzed using Pearson's correlation coefficient (*r*) using a one‐tailed directional hypothesis; ±0.1 signifies a small effect, ±0.3 a medium effect, and ±0.5 a large effect. To assess the main effects of loneliness, depression, and their interaction 2 (low and high loneliness) * 2 (low and high depression) full factorial ANOVAs were used. The dependent measures on the facial emotion recognition task were accuracy of identifying the emotion, reaction times for selecting the correct emotion, and which emotion was misattributed when presented with a neutral face. The factorial ANOVA was conducted as a two‐tailed hypothesis at a 0.05 significance level. Where partial eta squared (*η*²) is provided, 0.01 represents a small effect, 0.06 a medium effect, and 0.14 a large effect.

## 3 RESULTS

### 3.1 Initial screening: correlation between loneliness and depression for all screened participants

**FIGURE 1:**
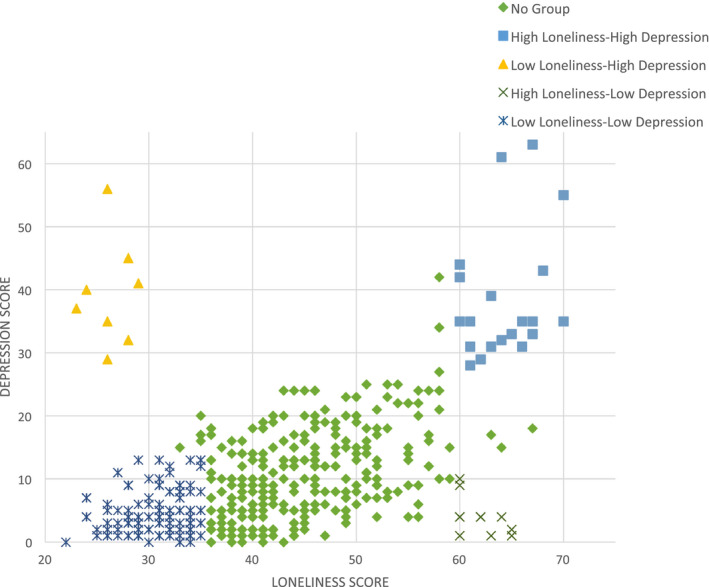
Pearson's correlation coefficient between loneliness (measured by the UCLA loneliness scale) and depression (measured by the Beck Depression Inventory) score; *n* = 502; *r* = .48; *p* < .001

There was a significant positive correlation between loneliness score and depression score, *r* = .48, *p* (one‐tailed) < .001. Thus, participants who reported higher levels of loneliness were also more likely to be depressed (Figure 1).

### 3.2 Facial emotion recognition task

#### 3.2.1 Response accuracy in the facial emotion recognition task

**FIGURE 2:**
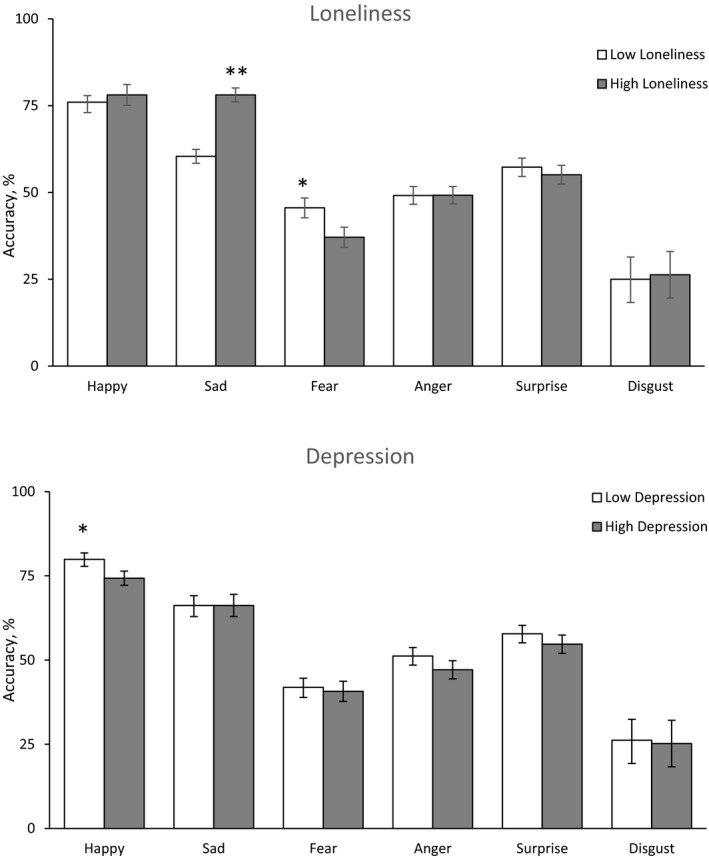
Mean accuracy (±*SD*) in recognizing the six facial emotions in participants that scored high in loneliness (a) or high in depression (b) (*p* < .05, **p < .05)

There was a significant effect of emotion type [*F* (5,73) = 67.50, *p* = .001] with happy being the most accurately identified emotion and disgusted being the least accurately identified emotion (see Table 2). Because of the significant effect of emotion type on accuracy, subsequent analysis used separate 2 (loneliness: high vs. low) * 2 (depression: high vs. low) factorial ANOVA's on each of the six emotions. When depression was held constant, loneliness was associated with increased accuracy for sad faces [*F* (1,73) = 7.02, *p* = .010, *η*² = 0.088; Figure 2a), and decreased accuracy for fearful faces, [*F* (1,73) = 4.89, *p* = .030, *η*² = 0.063; Figure 2a]. When loneliness was held constant, depression was associated with decreased accuracy in identifying happy faces [*F* (1,73) = 4.02, *p* = .038, *η*² = 0.058; Figure 2b). There were no statistically significant main effects for disgusted, angry, or surprised faces and no statistically significant interactions across any of the six emotions [in all cases *F* < 0.98, *p* NS].

**TABLE 2:** Means and Standard Deviations (*SD*) for the three outcome variables for each emotion type

	HL/HD (*n* = 21) Mean (*SD*)	LL/HD (*n* = 10) Mean (*SD*)	HL/LD (*n* = 11) Mean (*SD*)	LL/LD (*n* = 35) Mean (*SD*)
Happy Act	75.95 (8.84)	72.60 (13.06)	80.27 (10.44)	79.49 (11.30)
Sad Acc	72.67 (11.87)	59.80 (28.01)	71.55 (9.37)	60.91 (17.32)
Fear Acc	37.19 (18.12)	44.30 (7.33)	37.09 (14.60)	46.80 (15.83)
Angry Acc	45.90 (14.61)	48.30 (16.00)	52.27 (17.74)	50.14 (12.09)
Disgusted Acc	30.33 (40.23)	20.00 (42.16)	22.36 (34.08)	30.06 (31.62)
Surprise Acc	52.14 (14.74)	57.20 (12.24)	58.09 (12.38)	57.46 (15.03)
Happy RT (ms)	75.95 (8.83)	72.60 (13.06)	80.27 (10.44)	79.49 (11.30)
Sad RT (ms)	72.67 (11.87)	59.80 (28.01)	71.55 (9.37)	60.91 (17.32)
Fear RT (ms)	37.19 (18.12)	44.30 (7.33)	37.09 (14.60)	46.80 (15.81)
Angry RT (ms)	45.90 (14.61)	48.30 (16.00)	52.27 (17.74)	50.14 (12.09)
Disgusted RT (ms)	30.33 (40.23)	20.00 (42.16)	22.36 (34.08)	30.06 (31.62)
Surprise RT (ms)	52.14 (14.72)	57.20 (12.24)	58.09 (12.38)	57.46 (15.03)
Happy MB	0.25 (0.57)	0.40 (0.69)	0.00 (0.00)	0.59 (1.06)
Sad MB	4.25 (0.93)	4.00 (1.70)	4.00 (1.41)	2.12 (1.36)
Fear MB	0.06 (0.25)	0.20 (0.63)	0.25 (0.46)	0.53 (0.87)
Angry MB	0.94 (0.68)	0.80 (0.42)	1.00 (0.92)	1.35 (0.86)
Disgusted MB	0.38 (0.71)	0.50 (0.85)	0.50 (0.75)	1.00 (1.06)
Surprise MB	0.13 (0.32)	0.10 (0.31)	0.25 (0.70)	0.41 (0.79)

Abbreviations: Acc, accuracy; HL/HD, high loneliness and high depression; HL/LD, high loneliness and low depression; LL/HD, low loneliness and high depression; LL/LD, low loneliness and low depression; MB, misattribution bias; RT, reaction time.

#### 3.2.2 Reaction times in the facial emotion recognition task

There was a significant main effect of emotion type [*F* (5,73) = 10.21, *p* = .001] with reaction times being longest for happy and shortest for disgusted and fear (see Table 2). Because of these differences, subsequent analysis for each of the six emotions was conducted separately. There were no statistically significant main effects or interactions between loneliness and depression for any of the emotions on reaction times [in all cases *F* < 2.53, *p* NS].

#### 3.2.3 Misattribution of neutral faces in facial emotion recognition task

**FIGURE 3:**
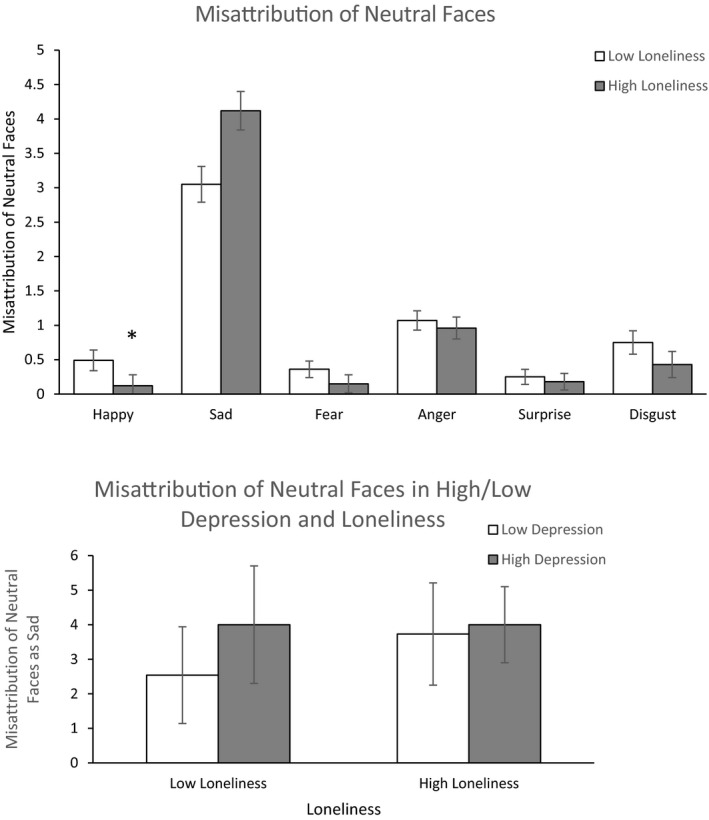
Main score (±*SD*) in (a) misattribution of neutral faces as happy in participants who are low or high in loneliness (b) interaction between loneliness and depression in the misattribution of neutral faces. High loneliness and high depression and both high loneliness/high depression resulted in the misattribution of neutral faces as sad. Low loneliness and low depression displayed a lower level of this misattribution bias

When depression was held constant, lonely individuals were significantly less likely to misattribute the emotion of happiness to neutral faces [*F* (1,56) = 4.605, *p* = .036, *η*² = 0.076; Figure 3a). There was also a significant interaction between loneliness and depression on the misattribution of neutral faces as sad [*F* (1,73) = 6.377, *p* = .014, *η*² = 0.080]. High loneliness and high depression and both high loneliness/high depression resulted in a comparable level of misattribution of neutral faces as sad. Only, those participants with low loneliness/low depression displayed a lower level of this misattribution bias. There were no main effects of loneliness and depression on classifying neutral faces as fearful, disgusted, angry, or surprised and no statistically significant interaction between loneliness and depression across happy, fearful, disgusted, angry, or surprised faces [in all cases *F* < 3.55, *p* NS].

## 4 DISCUSSION

In the present study, loneliness and depression scores were positively correlated, a finding, which replicates previous studies (Cacioppo, Hawkley, et al., 2006; Cacioppo, Hughes, et al., 2006; Mahon et al., 2006). This is the first study to demonstrate that this comorbidity is associated with shared deficits on a facial emotion processing task, specifically the misattribution of neutral faces as sad. This misattribution bias, however, was also found in participants who were only lonely and low in depression and those who were only depressed and low in loneliness. Therefore, these findings only partially support our hypothesis that comorbidity would enhance the effects of loneliness and depression. The depression finings, however, are consistent with the literature where mood‐congruent biases are consistently reported (Bourke et al., 2010; Elliott et al., 2011). FMRI studies focusing on the neural correlates of negative emotional processing biases in depression have identified hyperactivation in the amygdala, insula, parahippocampal gyrus, the visual face area, that is, the fusiform gyrus and the putamen (Stuhrmann et al., 2011). Functional connectivity studies have revealed that these mood‐congruent biases show reduced connectivity between the amygdala and the dorsal/supragenual anterior cingulate cortex (ACC) regions and increased connectivity between the amygdala‐subgenual ACC. It is highly likely that these neural mechanisms are also responsible for the comorbid effects of loneliness and depression on emotional face processing, and this should be tested in future research.

As well as highlighting the comorbid effects of loneliness and depression on emotional processing, loneliness was associated with increased accuracy in identifying sad faces. Lonely individuals also showed reduced accuracy in fearful facial emotion processing. The effects of loneliness on accurately identifying emotions conform with previous literature proposing dysfunctional processing and differing sensitivity to some facial emotional cues (Lodder et al., 2016). Notably, enhanced recognition of sad faces is consistent with the current hypothesis and further substantiates previous research findings of a negative processing bias within lonely individuals (Hawkley & Cacioppo, 2010). A decreased accuracy in correctly identifying fearful faces within lonely participants is not consistent with previous findings of increased accuracy of angry (Lodder et al., 2016) and fearful expressions (Vanhalst et al., 2017). Typically, these findings have been explained within the context of theories on loneliness of increased vigilance to signals of social rejection (Cacioppo & Hawkley, 2009; Gardner et al., 2005). However, our findings are not consistent with such a theoretical account of loneliness as fear is also likely to signal social rejection. The methodology used to present the emotional stimuli differed between the present study and Vanhalst et al. (2017), which may explain the difference in results. In the present study, fearful faces were presented for a period of 500 ms at different intensities and across the six different emotions in a counterbalanced order. In contrast, Vanhalst et al. (2017) presented fear as a short movie clip with the stimulus growing in intensity from neutral to full intensity. It is plausible that such a paradigm (Vanhalst et al., 2017) may be more accessible to assessing hyper vigilance to potentially threatening stimuli.

In contrast to the individual effects of loneliness, depression resulted in a decreased accuracy in the identification of happy faces. This finding supports previous research showing impaired discrimination accuracy and a negative processing bias away from happy faces with depression (Chan et al., 2009; Surguladze et al., 2004). Many researchers have suggested that this deficit in emotional processing offer support to cognitive accounts of depression and have been the target of antidepressant treatment (Bourke et al., 2010; Demenescu et al., 2010).

There are several limitations to the present study. One limitation was that using the cut‐offs we selected, that is, for depression a score between 25 and 63 on the BDI and for loneliness a score of 60 and 80 on the UCLA, we were presented with the unexpected difficulty in recruiting participants that were either only lonely (10/502) or only depressed (11/502), which resulted in differing sample sizes of the four experimental groups. Previous studies assessing social information processing in loneliness have varied in sample size; Vanhalst et al. (2017) had 170 adolescent participants, Lodder et al., (2016) a sample of 170 college students, Kanai et al.'s (2012) fMRI study had 108 participants and finally, Gardner et al., (2005) used a sample of 95 undergraduate students. Although our experimental design was unique and allowed for the teasing out of differential effects of loneliness and depression on emotional processing, this design and the high cut‐offs used for loneliness and depression also made recruitment to our experimental conditions difficult. We started testing participants on the emotional recognition task if they met the requirements of one of our four experimental groups. A total of one hundred and twenty‐six participants screened positive for the control group (low loneliness/low depression) and thirty‐five of these completed the emotional processing task. Recruitment to the high loneliness/high depression group led to the screening of twenty‐three participants with twenty‐one of these being tested on the emotional processing task. Recruitment to groups that were either only lonely or only depressed proved extremely challenging and thus the decision was made that once ten participants in each group were obtained, recruitment would terminate. In the end, we were recruited eleven participants with high loneliness only and ten participants with high depression only and to achieve these numbers we had to screen 502 participants. Despite these difficulties, the present study does reflect participants within the same sample who are either only depressed, only lonely or comorbid for loneliness and depression. We fully acknowledge that despite a very large sample size for the correlation, the differences seen in the emotional processing task need to be confirmed in future well‐powered studies with more consideration given to the impact of different cut‐offs for loneliness and depression on the results. To further add, we have recently completed an online study exploring the relationship between loneliness and depression and other risk factors for depression including stress and rumination. The preliminary analysis of this data suggests that individuals on the highest end of the depression spectrum as scored on the BDI do not tend to be low on loneliness. We are writing up these results for peer‐review and publication, and they may offer support to evidence cited in the Introduction that loneliness predicts depression rather than depression predicting loneliness (Hawkley et al., 2006).

## 5 CONCLUSIONS

The present study adds to growing evidence on the detrimental effects of loneliness and depression which are highly comorbid on emotional processing. The uniqueness of the study is that it is the first to demonstrate independent and comorbid effects of loneliness and depression on emotional processing within the same sample. However, as the misattribution of sadness to neutral expressions was seen not only in the comorbid group but also in those who were either only lonely or only depressed, our findings do suggest that in order to prevent detrimental outcomes associated with poor emotion processing both depression and loneliness need to addressed and treated.

## CONFLICT OF INTEREST

None declared.

### PEER REVIEW

The peer review history for this article is available at https://publons.com/publon/10.1002/brb3.2189.

## Data Availability

Extra data can be extracted by emailing the corresponding author SC.
